# A Novel Ultrasound Window for Simultaneous Botulinum Neurotoxin Injection Into Three Deep Leg Muscles in Ankle and Foot Spasticity Patterns

**DOI:** 10.7759/cureus.99783

**Published:** 2025-12-21

**Authors:** Diogo Portugal, Klemens Fheodoroff, Miguel Reis e Silva, Jorge Jacinto

**Affiliations:** 1 Service of Rehabilitation for Adults 3, Botulinum Toxin Clinic, Gait Analysis Laboratory, Alcoitão Medicine and Rehabilitation Centre, Lisbon, PRT; 2 Neurorehabilitation Department, Gailtal-Klinik, Hermagor-Pressegger See, AUT; 3 Health and Performance Department, Sport Lisboa e Benfica, Lisbon, PRT

**Keywords:** botulinum toxins, interventional, lower extremity, muscle spasticity, ultrasonography

## Abstract

Ankle and foot conditions with muscle overactivity often require botulinum neurotoxin (BoNT) injections into the flexor digitorum longus (FDL), tibialis posterior (TP), and flexor hallucis longus (FHL) muscles. This manuscript introduces a novel ultrasound (US) window for simultaneous BoNT injections into these muscles to improve efficiency and patient comfort in treating ankle and foot spasticity. A novel posterior approach using a transverse US probe placement on the distal leg was developed in patients requiring BoNT injections for lower limb muscle overactivity. This technique modifies traditional probe handling to achieve a horizontal alignment of TP, FDL, and FHL within the US window, facilitating simultaneous visualization and a single skin puncture three-muscle in-plane injection. This single US window allows concurrent visualization of the TP, FDL, and FHL. It may facilitate more precise needle and injectate placement. The approach reduces the number of skin punctures. It may also improve procedural efficiency and patient comfort. This technique offers a practical option for BoNT injections into deep posterior leg muscles. It could be considered for routine clinical use in managing ankle and foot muscle overactivity.

## Introduction

Botulinum neurotoxin (BoNT) is a pivotal treatment option for managing muscle overactivity secondary to upper motor neuron lesions, such as stroke, cerebral palsy, multiple sclerosis, traumatic brain injury, and spinal cord injury. Spastic muscle overactivity may, over time, lead to soft-tissue changes. This includes contracture, atrophy, loss of sarcomeres, accumulation of intramuscular connective tissue, increased fat content, and degenerative changes at the myotendinous junction, which in turn can modify muscle architecture and position [1]. These alterations may contribute to a mismatch between external anatomical landmarks and the actual location of the spastic muscles, making accurate needle placement more challenging [2-7].

Among the various clinical patterns of lower limb spasticity, ankle and foot dynamic deformities are particularly frequent, especially equinovarus and flexed (claw) toes, postures/deformities, which often require BoNT treatment. Given that the efficacy of BoNT injections depends significantly on precise target muscle localization and intramuscular distribution of the toxin, optimization of guidance techniques is crucial. Several studies have shown that landmark-based manual needle placement may be inaccurate in spastic patients when compared with ultrasound (US)-guided approaches, leading to suboptimal localization of the target muscles [2-7]. Consequently, US guidance has gained a central role in improving the accuracy and safety of BoNT injections for common spastic patterns, including equinovarus foot [4-7].

However, traditional US-guided protocols for ankle and foot muscles frequently rely on multiple injection sites and windows for muscles such as the flexor digitorum longus (FDL), tibialis posterior (TP), and flexor hallucis longus (FHL). This increases procedural complexity, treatment time, patient discomfort, and potential iatrogenic risk. In particular, deep muscles of the posterior compartment often require separate approaches (anterolateral, medial, and posteromedial), each with its own limitations in terms of needle visualization and proximity to neurovascular structures.

To address these challenges, the present article describes a single posterior leg US window designed to enable simultaneous visualization and injection of FDL, TP, and FHL using an in-plane technique and a single skin puncture. Building on current anatomical and ultrasonographic knowledge, this approach aims to simplify the injection procedure, enhance patient comfort, and potentially improve safety and efficiency in the management of ankle and foot muscle overactivity. This technique was previously presented by the first author as a poster, including a brief oral presentation, at the 24th European Congress of Physical and Rehabilitation Medicine (ESPRM) in Ljubljana (Slovenia) on April 25, 2024.

## Materials and methods

The authors utilized their experience with US-guided procedures in BoNT clinics, totaling 37 years overall. The US images in this manuscript were acquired using a 5.0-10.0 MHz linear transducer (Siemens ACUSON NX3TM, Siemens Medical Solutions) and a 3-12 MHz linear array probe (VScan AirTM, GE Healthcare).

Patient position and US technique

**Figure 1:**
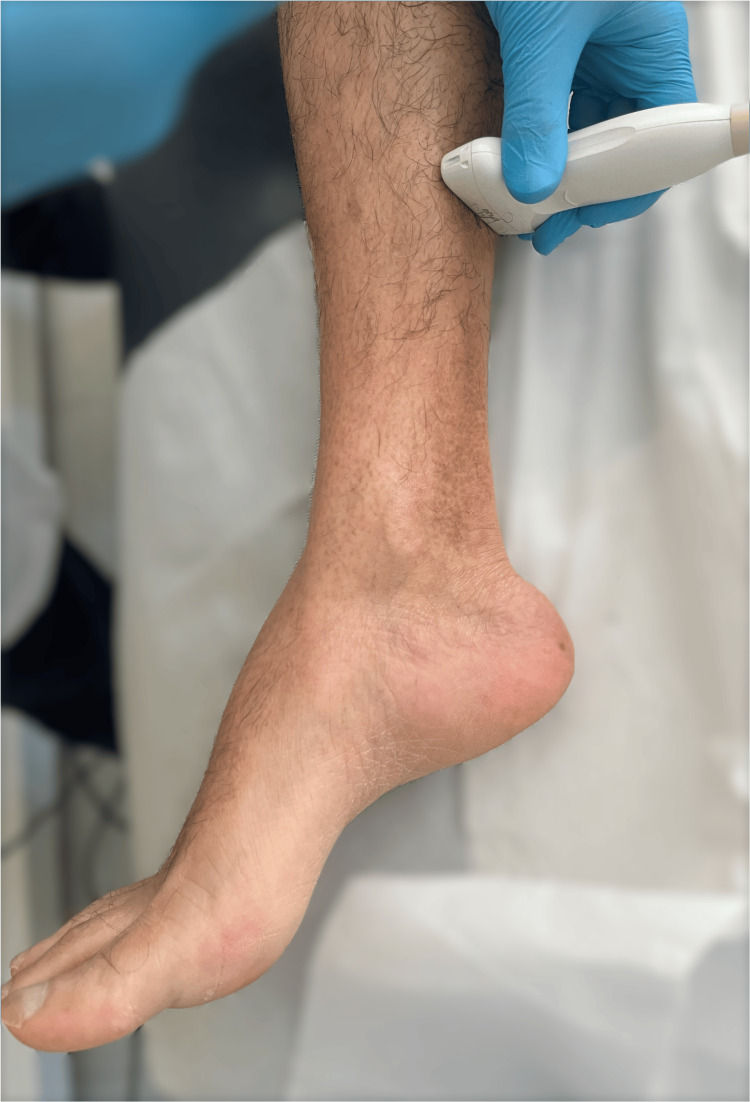
Patient position (supine) and probe placement

**Figure 2:**
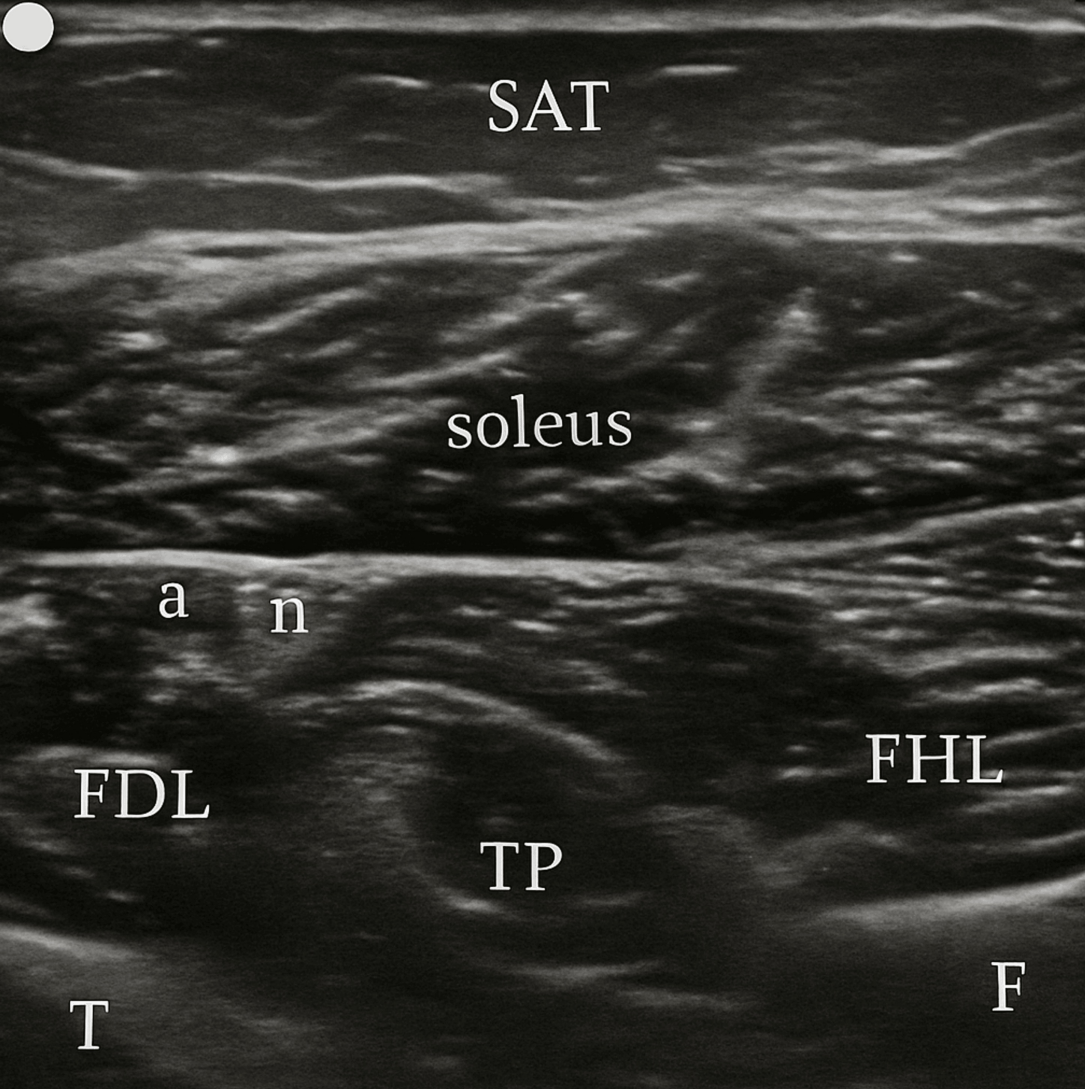
The proposed US window US: ultrasound; SAT: subcutaneous adipose tissue; T: tibia; F: fibula; a: posterior tibial artery; n: tibial nerve

**Figure 3:**
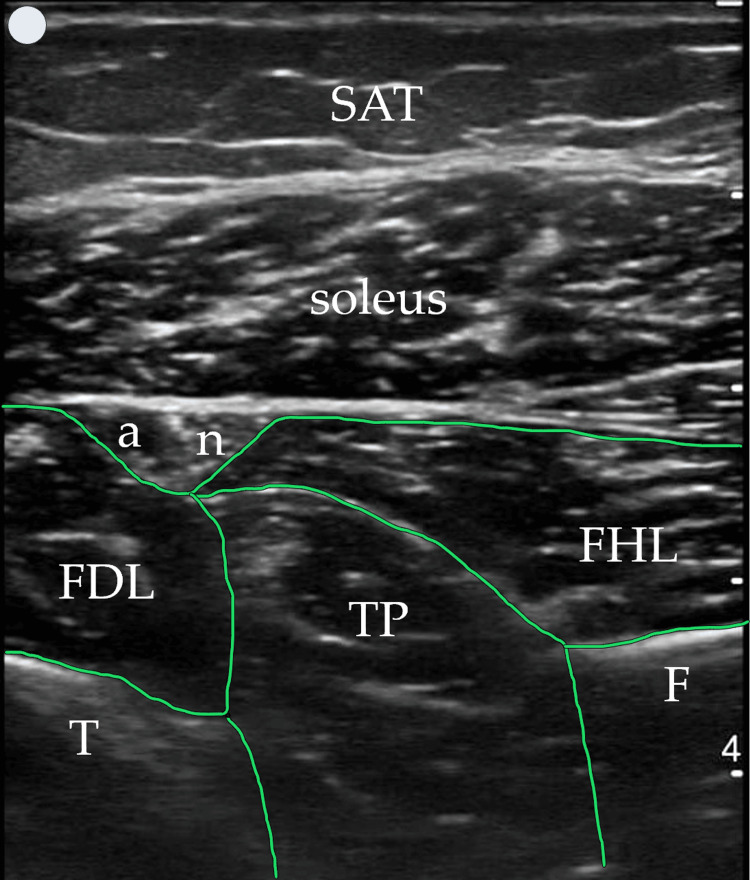
The proposed US window-target muscles highlighted in green US: ultrasound; SAT: subcutaneous adipose tissue; t: tibia; f: fibula; a: posterior tibial artery; n: tibial nerve

The patient is in a comfortable and standard supine position, and a transverse US probe placement is utilized on the posterior middle and distal third of the leg (Figure 1), providing simultaneous visualization of FDL, TP, and FHL muscles (Figures 2-3). Traditional clinical practices often use a posteromedial approach for FDL and FHL injections and an anterolateral or posteromedial approach for TP. These approaches typically position the FDL and FHL muscles obliquely and require out-of-plane TP muscle injection, hampering simultaneous visualization and optimal needle guidance.

**Figure 4:**
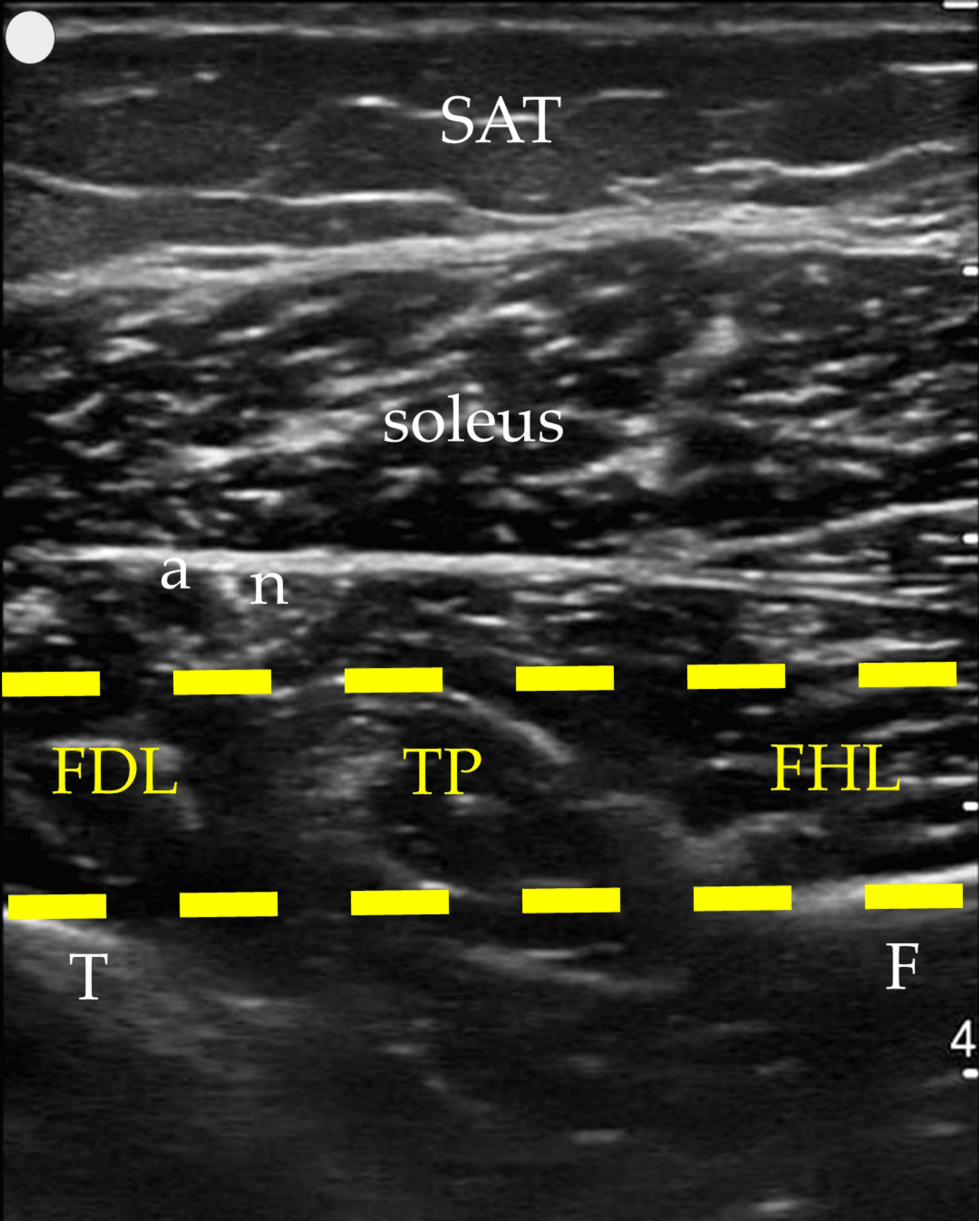
The proposed US window-needle “tunnel” highlighted in yellow, within the limits “ceiling” (soleus) and “ground” (tibia and fibula) US: ultrasound; SAT: subcutaneous adipose tissue; T: tibia; F: fibula; a: posterior tibial artery; n: tibial nerve

In contrast, the posterior approach we describe and propose enhances muscle visibility by modifying probe handling. By slightly elevating the anterior end of the probe (a minor standoff technique) and applying gentle pressure with the posterior end, the muscles align more horizontally within the US window. This adjustment creates an optimal imaging plane, where the tibia and fibula serve as the base or "ground," and the overlying soleus muscle acts as the "ceiling," creating a needle “tunnel” between these structures where the three target muscles can be injected (Figure 4). This positioning not only facilitates a clearer view of the muscle structures but also allows for more precise in-plane injections in all three muscles if necessary. The needle's entry and progression can be continuously monitored, significantly enhancing both the safety and efficacy of the injection process. Figures 2-4 were acquired using a 3-12 MHz linear array probe (VScan AirTM, GE Healthcare) from a patient with Heckmatt scale [8] Grade 1/4 muscle echogenicity.

Injection technique

**Figure 5:**
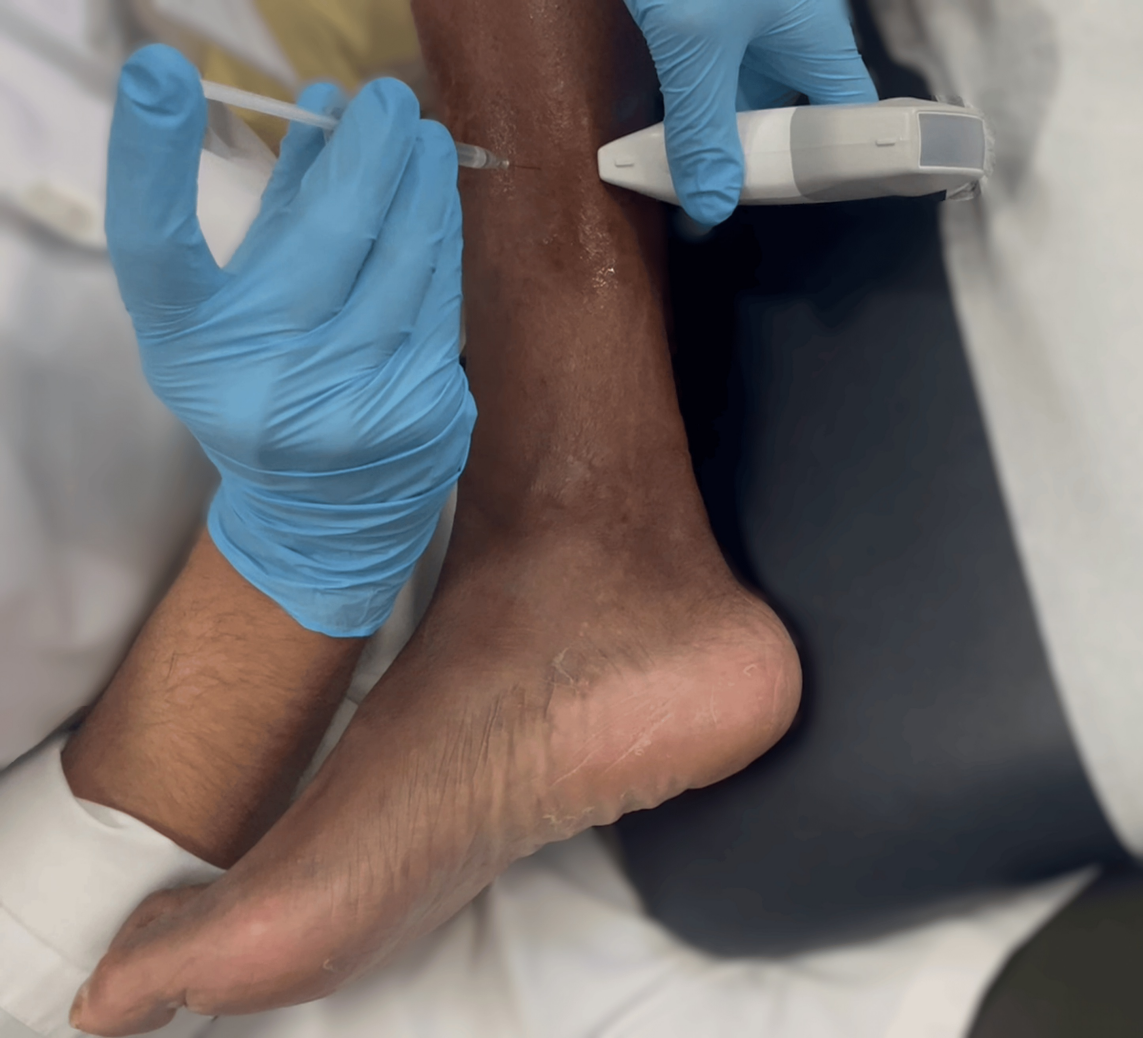
Injection site

**Figure 6:**
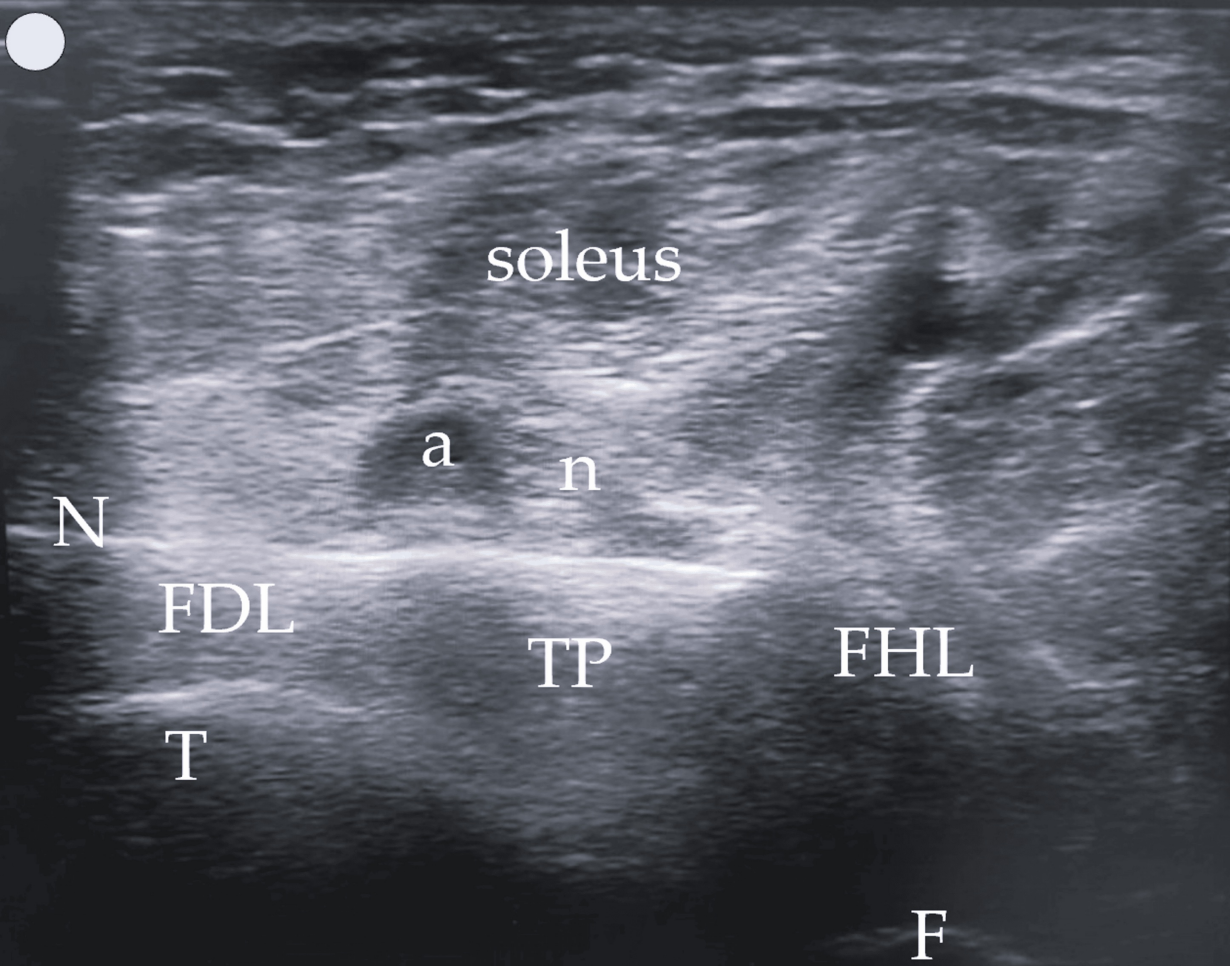
In-plane needle visualization into the FHL FHL: flexor hallucis longus; T: tibia; F: fibula; a: posterior tibial artery; n: tibial nerve; N: needle (27 G, 40 mm needle)

The injection site (Figure 5) is positioned medially, adjacent to the tibia. Using an in-plane technique (with the needle parallel to the transducer, Figure 6), the needle is first advanced into the FHL muscle for BoNT delivery, followed by the TP and FDL muscles as the needle is withdrawn toward the surface. This method allows for a single-entry point for BoNT injection into all or up to three muscles, reducing the number of skin punctures and patient discomfort. Figure 6 was acquired using a 5.0-10.0 MHz linear transducer (Siemens ACUSON NX3TM, Siemens Medical Solutions) from a patient with Heckmatt scale [8] Grade 3/4 muscle echogenicity.

## Results

Advantages of the US window

The posterior leg US window offers a comprehensive view of the three deep leg muscles in a single plane, suggesting four significant advantages:

Concurrent Muscle Assessment

**Figure 7:**
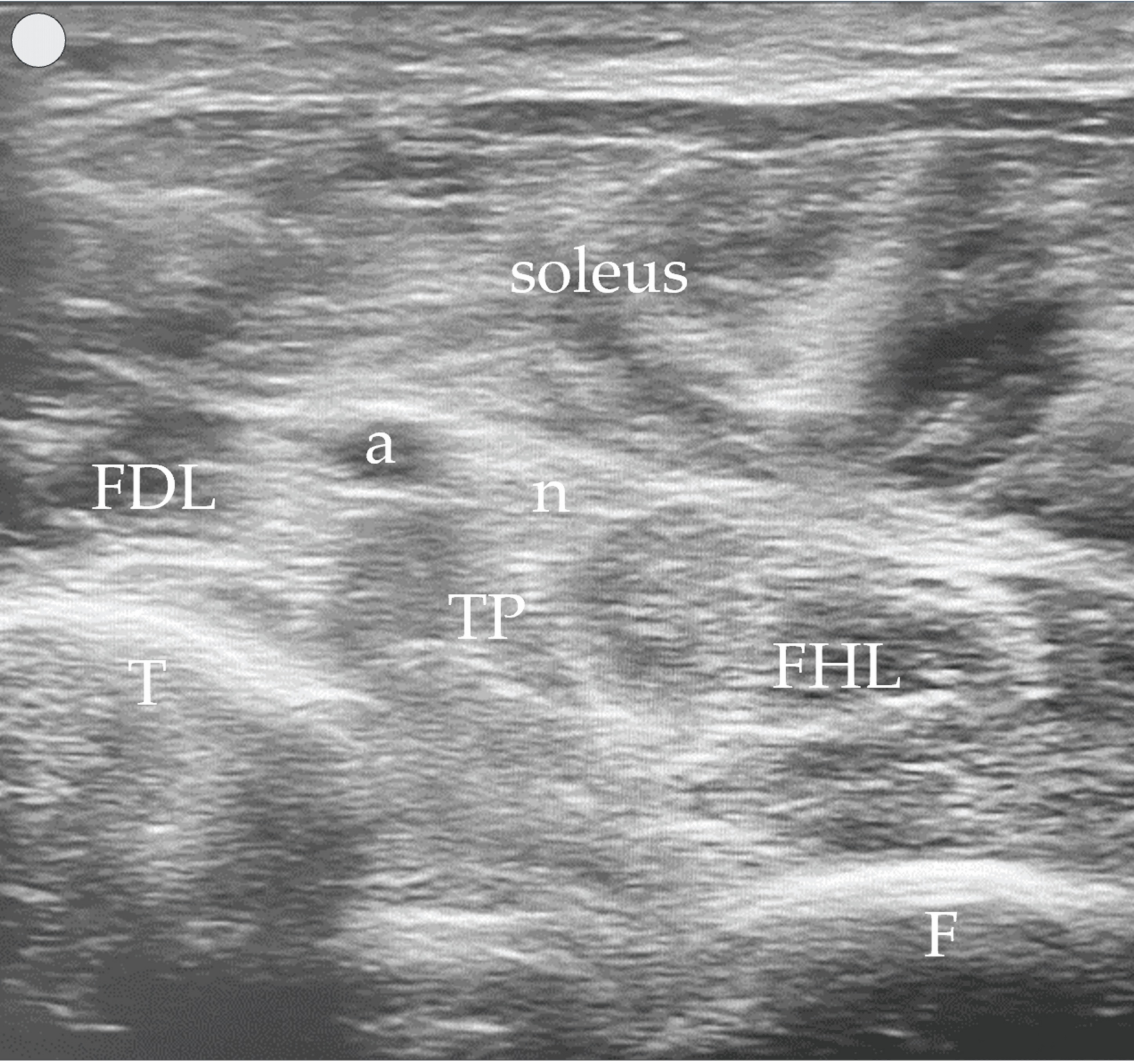
Muscle echogenicity assessment-Grade 3/4 Heckmatt scale T: tibia; F: fibula; a: posterior tibial artery; n: tibial nerve

Precise localization and assessment of muscle position, depth, size, and architecture. This approach enables the simultaneous evaluation of muscle echogenicity (Figure 7) using the Heckmatt scale [8], together with hypertrophy and atrophy in these three muscles. The combined assessment can indicate which muscle is predominantly overactive and whether chronic structural changes are present, thereby informing muscle selection and expectations of response to BoNT. Figure 7 was acquired using a 5.0-10.0 MHz linear transducer (Siemens ACUSON NX3TM, Siemens Medical Solutions).

Simultaneous Injection

A single US window with in-plane needle visualization allows BoNT injection into TP, FDL, and FHL through one skin entry point, reducing skin punctures from three to one (or from four to two when a two-point TP technique is used to optimize toxin distribution), thereby potentially decreasing patient discomfort and iatrogenic risk.

Improved Needle Visualization

The in-plane visualization of the needle facilitates continuous monitoring of the needle, target muscles, innervation zones, and vascular structures to be avoided, warranting safety and precision.

Enhanced Injectate Visualization

The single US window delivers excellent visualization of the injectate within the target muscle tissue, ensuring accurate placement and potentially enhancing treatment outcomes.

## Discussion

Multiwindow techniques for the FDL, TP, and FHL [4] typically require multiple probe positions and skin punctures, whereas a single-window strategy may reduce the total number of punctures and simplify workflow. In usual clinical practice, FDL, TP, and FHL require several injections (at least four injections) and US windows (at least two to three windows): FDL typically requires one injection, using an out-of-plane medial/posteromedial approach; TP often necessitates two injection sites, commonly using an out-of-plane anterior/anterolateral or medial approach; and FHL generally involves one injection, with an out-of-plane posteromedial approach.

In contrast, the single posterior leg US window could be considered a lower-burden approach, as it reduces the number of injection points and needle redirections and may therefore lessen the cumulative procedural risk. Furthermore, this approach allows for the simultaneous assessment of muscle structure, providing valuable information for treatment decisions.

Cadaveric studies mapping intramuscular nerve distribution have described innervation zones for FDL and FHL predominantly in the distal segments of the muscle belly (approximately 60-70% and 50-80% of muscle length, respectively), which is consistent with the level where the probe is positioned in the present US window. In contrast, the main motor point area of TP has been reported more proximally, around 20-30% of muscle length [9]. Although this apparent discrepancy might be seen as a limitation of the proposed window for TP targeting, it should be interpreted in the context of both anatomical access and the clinical setting of spastic muscle overactivity. Targeting TP at 20-30% of its length generally requires an anterolateral leg window, with needle advancement across the interosseous membrane between the tibia and fibula. This approach is usually performed out-of-plane, which reduces needle tip visualization and may increase the theoretical risk to anterior compartment neurovascular structures. In anticoagulated patients, any bleeding-related complication could also theoretically increase the risk of acute compartment syndrome [10]. By contrast, the posterior window used in this technique allows in-plane needle visualization, avoids transfixing the interosseous membrane, and provides a more controlled access to the deep compartment.

Spastic muscle overactivity may lead, over time, to substantial structural changes within the muscle and surrounding soft tissues, including contracture, atrophy, loss of sarcomeres, accumulation of intramuscular connective tissue, increased fat content, and degenerative changes at the myotendinous junction [1]. These local anatomical modifications can alter muscle architecture and position and thereby contribute to a mismatch between surface landmarks and the actual location of the spastic muscles.

Several studies have demonstrated that, in patients with chronic spasticity, manual needle placement based on palpation and anatomical landmarks alone may be inaccurate when compared with US-guided localization. This has been shown for forearm flexor muscles [2,3], for the medial approach to TP in equinovarus foot [5], and for gastrocnemius injections in spastic equinus [6,7]. In these works, US guidance improved the accuracy of BoNT injection site localization and, in some cases, was associated with better clinical or procedural outcomes compared with landmark-based or electrical-stimulation guidance alone.

Therefore, the use of ultrasonography has gained importance in improving BoNT injections for managing some of the most frequent spastic muscle overactivity patterns, such as equinovarus foot [5-7]. On this basis, it is plausible that the soft-tissue contracture process due to spastic paresis may also alter the anatomical position of other key structures relevant for BoNT delivery, including the relative location of intramuscular nerve branches and terminal endings. In this context, strict reliance on motor point maps derived from nonspastic cadaveric specimens may not fully reflect the functional anatomy of chronically spastic muscles in vivo.

The posterior single-window approach described in this manuscript fits within this ultrasonographic paradigm: it enables real-time visualization of TP, FDL, and FHL in their actual, possibly remodeled positions, and allows for a single in-plane needle trajectory to reach all three muscles with high precision. Even if the injection site for TP does not coincide exactly with the proximally described innervation zone, the combination of adequate intramuscular spread of the injectate, safe needle trajectory, and optimized procedural efficiency may render this approach clinically effective and attractive for routine practice when treating ankle and foot muscle overactivity.

Limitations

This article is descriptive and does not provide comparative or quantitative outcome data. Accordingly, potential advantages such as improved efficiency, reduced discomfort, and reduced iatrogenic risk should be interpreted as hypotheses supported by procedural rationale rather than empirically proven outcomes. In addition, the technique may be operator-dependent and influenced by patient-specific factors (e.g., anatomy, degree of spasticity, body habitus). Prospective studies comparing multiwindow and single-window protocols, including measures of procedure time, number of skin punctures, patient-reported discomfort, technical success, and adverse events, are needed to confirm these potential benefits.

## Conclusions

The use of a single US window for BoNT injection into the FDL, TP, and FHL muscles is a useful addition to the clinical toolkit for treating ankle and foot spasticity. This technique may enhance clinical efficiency and improve patient comfort. It may also facilitate precise drug delivery and potentially reduce procedural risk, supporting its consideration for routine clinical practice.
